# Tuning multispectral fluorescence quantum dot–based identification of short-length amyloid β peptides by applying Cu(II) ions

**DOI:** 10.1007/s00604-024-06764-9

**Published:** 2024-10-26

**Authors:** Klaudia Głowacz, Weronika Tokarska, Anita Olechowska, Nina E. Wezynfeld, Patrycja Ciosek-Skibińska

**Affiliations:** Chair of Medical Biotechnology, Faculty of Chemistry, Warsaw University of Technology, Noakowskiego 3, 00-664 Warsaw, Poland

**Keywords:** Chemical tongue, Competitive assay, Quantum dots, Multispectral fluorescence, PLS-DA, Amyloid β

## Abstract

Currently available methods for detecting amyloid β (Aβ) derivatives are mainly dedicated to determining the long forms Aβ_1-42_ and Aβ_1-40_. At the same time, the number of physiologically occurring Aβ analogs is much higher, including those truncated at the N- and C-termini. Their identification using standard methods is challenging due to the structural similarity of various Aβ analogs, but could highly benefit from both biomarkers discovery and pathophysiological studies of Alzheimer’s disease. Therefore a “chemical tongue” sensing strategy was employed for the detection of seven Aβ peptide derivatives: Aβ_1-16_, Aβ_4-16_, Aβ_4-9_, Aβ_5-16_, Aβ_5-12_, Aβ_5-9_, Aβ_12-16_. The proposed sensing system is based on competitive interactions between quantum dots, Cu(II) ions, and Aβ peptides, providing unique fluorescence fingerprints useful for the identification of analytes. After carefully evaluating the Aβ sample preparation protocol, perfect determination of all studied Aβ peptides was achieved using partial least square–discriminant analysis (PLS-DA). The developed PLS-DA models are characterized by excellent accuracy, sensitivity, precision, and specificity of analyte determination, emphasizing the potential of the proposed sensing strategy.

**Graphical abstract:**

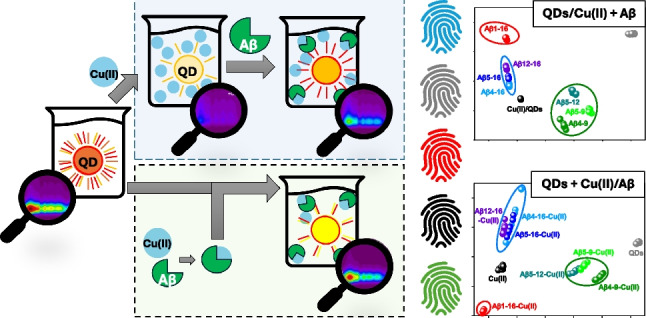

**Supplementary Information:**

The online version contains supplementary material available at 10.1007/s00604-024-06764-9.

## Introduction

Alzheimer’s disease (AD) is the most common cause of dementia. It typically affects people age 65 or older and is recognized as the fifth leading cause of death in the USA. The situation is getting worse every year with the increasing number of older people and the lack of significant breakthroughs in AD treatment. Currently available drugs only moderate symptoms (donepezil, rivastigmine, galantamine, memantine) or aim to slow the rate of AD progression (aducanumab and lecanemab), but with questionable effectiveness and cause numerous side effects [1]. Another challenge is associated with the diagnosis of Alzheimer’s disease, which nowadays includes cognitive, functional, and behavioral tests; identification of AD biomarkers; and brain imaging. The assessment based on symptoms could be misleading as even 30% of individuals whose behavior seems to be typical for this disease do not reflect characteristics for AD changes in the brain [2]. These AD-related changes usually correspond to aggregates mostly composed of amyloid β (Aβ) peptides. Thus, the level of Aβ_1-42_ in cerebrospinal fluid (CSF) is typically measured to confirm AD. However, there are numerous forms of Aβ peptides, which differ in the number of amino acids. Their sequences are usually truncated at N- and/or C-termini compared to Aβ_1-42_ [3]. Consequently, they present various properties in processes strictly related to AD, such as aggregation and formation of species catalyzing reactive oxygen species (ROS) [4]. They could also serve as potential AD biomarkers as their amounts varied in the brains of healthy and AD individuals. Expanding the pool of determined AD-related biomarkers is very challenging with usually employed immunoassays [3, 5] due to a close structural similarity and, in the case of short-length forms, too small size of the potential epitope [5, 6]. Therefore, there is an urgent need to develop novel methods for discriminating such analogous compounds.

A potentially attractive strategy for the identification of such structurally similar compounds is the “chemical tongue” sensing approach (also called “differential sensing” or “pattern-based sensing”) [7, 8]. A major advantage of chemical tongues is their high efficiency in complex analyte sensing, which usually results from using multiple cross-reactive receptors able to recognize and generate distinct response patterns for each investigated analyte. The resulting chemical “fingerprints” produced by an array of receptors can then be processed with various machine learning algorithms to recognize single analytes or their mixtures [7, 9]. The replacement of traditional specific sensing (relying on a “lock and key” recognition mechanism) with a chemical tongue approach does not involve the time-consuming and laborious design of receptors for each investigated analyte, which can be advantageous when analyzing mixtures of compounds. The further simplification of the “chemical tongue” system is also possible through the use of a single, differentially interacting receptor element and advanced detection techniques, producing multidimensional optical information [10, 11]. In addition, the same chemical tongue system often detects multiple analytes with one or relatively few recognition elements, which makes pattern-based sensing methods widely adopted in the analytical community [9].

Recently, several chemical tongue methods were proposed as a promising strategy for the detection of Aβ peptides, where nanomaterials such as manganese dioxide nanozymes [12], poly(amidoamine) dendrimers [13], and commercially available fluorescent dyes coupled with graphite oxide (GO) [14] or polymers [15] were used for the design of an array. However, the presented methods focused only on detecting two Aβ isoforms (Aβ_1-40_ and Aβ_1-42_) and distinguishing different forms of their aggregates. Considering the variety of possible, shorter amyloid β species that may contribute to Alzheimer’s disease development process, and on the other hand, the design of treatment programs, novel methods for their recognition are highly desirable.

To fill this gap, we decided to test the possibility of using quantum dots (QDs) for the identification of short-length Aβ peptides [10]. However, instead of using an array of receptors, we showed that selected Aβ peptides differentially interact with the surface of thiomalic acid–capped CdTe quantum dots only and this interaction can be captured with signal enrichment provided by multispectral fluorescence (excitation-emission matrix fluorescence spectroscopy). This way, excellent discrimination was obtained for seven amyloid β analogs: Aβ_1-16_, Aβ_4-16_, Aβ_4-9_, Aβ_5-16_, Aβ_5-12_, Aβ_5-9_, Aβ_12-16_. While the detection of Aβ_4-16_, Aβ_5-16_, and Aβ_5-9_ in binary and ternary mixtures performed by QDs-based chemical tongue provided perfect 100% accuracy for the two studied peptides (Aβ_4-16_ and Aβ_4-16_), for the third one (Aβ_5-9_), however, it was slightly lower (97.9%). As chemical tongue recognition is based on cross-reactive interactions, further expansion of the library of analytical targets could demand further enrichment of accessible analytical information for satisfactory recognition capabilities. For this purpose, competitive interactions can be employed—such competitive assay strategies were successful in differential sensing of various proteins, bacteria, fungi, and normal or cancerous cells [16–19].

To this date, quantum dots have been extensively used for metal ion detection, based on changes in their fluorescence signal resulting from various photophysical mechanisms between the nanomaterial’s surface and analyte [20]. On the other hand, excellent and differentiated coordination properties of Aβ peptides toward transition metal ions as well as interactions of Aβ peptides with QDs [10] suggest that competitive assay based on ternary system (QDs, metal ions, Aβ peptides) could potentially provide higher accuracy and sensitivity of the chemical tongue-based detection compared to the binary system (QDs, Aβ peptides [10]). Aβ peptides can bind Cu(II) ions [21], which, in turn, can induce the change in the fluorescence response of quantum dots capped with simple anionic thiol ligands (e.g., 3-mercaptopropionic acid, MPA) [22, 23]. The Cu(II) binding sites occur exclusively at the N-terminal part of the Aβ peptide sequence, and the resulting structure and stability of Cu(II)/Aβ complexes depend strictly on the number and position of His residues. For example, one or two of three available His residues (at the 6th, 13th, and 14th position) could be involved in the Cu(II)/Aβ_1-16_ structure due to a dynamic equilibrium between two components observed at pH 7.4 [3]. In contrast, the truncated forms Aβ_4-16_ and Aβ_5-16_ comprise the His residue at the third (His-3 motif) or the second position (His-2 motif), respectively, allowing for forming very stable Cu(II) complexes. In addition, there is another, much weaker Cu(II) binding site in the further sequence of Aβ_4-16_ and Aβ_5-16_ involving two adjacent His residues (the *bis*-His motif) [24, 25]. The His-2 and His-3 motifs could also be combined as in the Aβ_12-16_ sequence, where the Cu(II) ion could switch between two species of high Cu(II) affinity [26].

This plentiful scheme of interactions between Cu(II) ions and Aβ peptides, Aβ peptides and QDs, and finally Cu(II) ions and QDs, additionally supported by multivariate characterization of all of these interactions by multispectral fluorescence, could provide a powerful detection system whose performance could be expected to be superior compared to the direct detection approach. Thus, the aim of this work was to test this research hypothesis—we studied the performance of the competitive chemical tongue system based on QDs and Cu(II) ions in the recognition of seven close analogs of Aβ peptides, applying two different assay procedures, for further determination and comparison of figures of merits respective for this identification task.

## Experimental section

### Reagents and materials

Quantum dots with CdTe core and thiomalic acid surface ligand (QDs), core diameter of 1.5 nm, and *λ*_max_ of 510 nm were obtained from PlasmaChem GmbH (Berlin, Germany). Amyloid β (Aβ) peptides were synthesized according to the Fmoc procedure and obtained from the Institute of Biochemistry and Biophysics PAS (Warszawa, Poland). The concentration of the Aβ stock solutions was determined by UV–Vis spectroscopy measurements, using an extinction coefficient related to Tyr (*ε*_276–296_ = 1410 cm^−1^ M^−1^) for Aβ_1-16_, Aβ_4-16_, Aβ_5-16_, and Aβ_5-12_ or by UV–Vis titrations of the peptide solution with Cu(II) for A_β4-9_, Aβ_5-9_, and Aβ_12-16_. *N*-(2-hydroxyethyl) piperazine-*N*′-(2-ethane sulfonic acid) (HEPES), copper(II) nitrate, and sodium hydroxide were obtained from Sigma-Merck (Poznań, Poland). Milli-Q water was used for the preparation of all aqueous solutions. All reagents were used as received.

### Sample preparation

Two protocols were applied during the preparation of QDs/Cu(II)/Aβ mixtures for multispectral fluorescence measurements. In the first one, Aβ peptides were added to previously prepared QDs/Cu(II) suspensions (1st approach: QDs/Cu(II) + Aβ). In the second one, the previously prepared Cu(II)/Aβ mixtures were added to QDs (2nd approach: QDs + Cu(II)/Aβ). The details of the sample preparation for those two approaches are given below.

#### 1^st^ approach: QDs/Cu(II) + Aβ

The first step was to prepare the mixture of QDs and Cu(II) ions (QDs/Cu(II)) by mixing stock solutions of QDs, Cu(II) ions, and 50 mM HEPES buffer pH 7.4 in a tightly sealed vial. The final concentration of QDs was 25 µg/mL, and Cu(II) ions was 4 µM. Then, the as-prepared mixture was incubated at ambient conditions for 60 min and pipetted into a 96-well plate (198 µL per well). Finally, 2 µL stock solution of each Aβ peptide in deionized water was added to the Cu(II)/QDs mixture reaching the concentration of 100 µM and Aβ:Cu(II) molar ratio of 25:1. The control sample of QDs was prepared by adding 198 µL of nanocrystal’s solution in HEPES buffer (50 mM, pH 7.4) and 2 µL of deionized water. The control samples of QDs/Cu(II) without peptides were prepared by adding 198 µL of QDs/Cu(II) mixture solution and 2 µL of deionized water. Each type of sample was prepared in eight independent replications. In this approach, samples were subjected to multispectral fluorescence measurements immediately after their preparation.

#### 2^nd^ approach: QDs + Cu(II)/Aβ

First, the mixture of Cu(II) ions and Aβ peptides was prepared by adding the stock solutions of Aβ peptide and Cu(II) ions to deionized water reaching final Cu(II) and Aβ concentrations of 200 µM and 5 mM, respectively. The pH value of these solutions was adjusted to 7.4 with concentrated Na(OH). Then, 196 µL of QD solution in 50 mM HEPES pH 7.4 was pipetted into a 96-well plate followed by 4 µL of respective Cu(II)/Aβ peptide mixture solutions. The final concentration of QDs in the well was 25 µg/mL, Cu(II) ions 4 µM, and Aβ peptide 100 µM, while Aβ:Cu(II) molar ratio of 25:1 was maintained. The control samples of QDs were prepared by adding 196 µL of QDs solution and 4 µL of deionized water. The control samples of QDs with Cu(II) ions were also included and prepared by adding 196 µL of QDs, 2 µL of Cu(II) ions, and 2 µL of deionized water to achieve predetermined conditions of concentrations of individual reagents in the measured sample. As-prepared samples were incubated for 60 min in a microplate reader before the collection of excitation-emission matrices. Each type of sample was prepared in eight independent replications.

### Multispectral fluorescence measurements

Excitation-emission matrices (EEM) were acquired with Synergy Neo2 Hybrid Multi-Mode Microplate Reader fluorescence spectrometer (BioTek Instruments, Inc., Winooski, VT, USA), using a handwritten protocol relying on recording consecutive emission spectra at increasing excitation wavelengths. Before the EEM measurements, the samples prepared in UV-Star 96-well plates (Greiner Bio-One GmbH, Kremsmünster, Austria) were mixed for 1 min. The emission spectra were recorded by changing the excitation wavelengths from 250 to 500 nm, with ∆*λ*_ex_ of 10 nm. To avoid 1st-order Rayleigh scattering, the range of the recorded emission spectra depended on the excitation wavelength at which the spectrum was acquired. Thus, for *λ*_ex_ ∈ (250 nm, 430 nm), the emission was recorded in the range of 450–700 nm, whereas for *λ*_ex_ ∈ (440 nm, 500 nm), fluorescence spectra were acquired at *λ*_em_ ∈ (*λ*_ex_ + 20 nm, 700 nm). All emission spectra were registered with a constant ∆*λ*_em_ of 1 nm. All experiments were performed at room temperature.

### Data analysis

The conducted multispectral fluorescence measurements resulted in a collection of 26 emission spectra per sample, which were further arranged in EEMs of 26 × 251 (*λ*_ex_ × *λ*_em_) each. Unfolded principal component analysis (PCA) and unfolded partial least square–discriminant analysis (PLS-DA) were used to compare Aβ peptide discrimination capabilities using investigated sample preparation protocols (1st approach: QDs/Cu(II) + Aβ and 2nd approach: QDs + Cu(II)/Aβ). Therefore, each excitation-emission matrix was unfolded into a data vector by combining the excitation and emission mode (1 × [(*λ*_ex_ × *λ*_em_)]). The resulting data vector describing each sample was 1 × 6246 (missing data resulting from the measurement procedure were omitted). The data matrices applied to PCA were 72 × 6246 (samples × [(*λ*_ex_ × *λ*_em_)]). Before PLS-DA modeling, the samples were randomly split in a ratio of 5:3 into train set and test set, which were used for model calibration and independent validation, respectively. Before validating models with an independent test set, a cross-validation of Venetian blind was performed. The optimal number of latent variables (LVs) describing the PLS-DA model was selected based on the minimalization of classification error of cross-validation. Mean centering was applied as the data pre-treatment step. Chemometric analysis was performed in Solo 9.1 (Eigenvector Research, Inc., Manson, USA). Figures were created in Origin (OriginLab Corporation, Northampton, MA, USA) software.

## Results and discussions

### Fluorescence response of QDs to Cu(II) ions

**Fig. 1:**
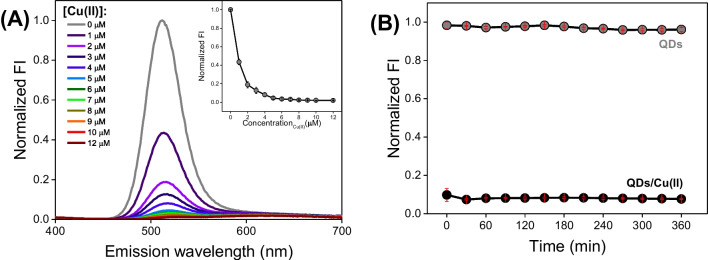
**A** Fluorescence titrations of 25 µg/mL QDs with Cu(II) ions in 50 mM HEPES buffer at pH 7.4. The presented fluorescence spectra were prepared based on data from three replicates for each sample type (*λ*_ex_ : 290 nm, *λ*_em_ : 310–700 nm). **B** Time dependence of normalized fluorescence intensities of QDs and QDs/Cu(II) mixture prepared based on emission spectra acquired at *λ*_ex_ : 290 nm, *λ*_em_ : 310–700 nm in respective time points

We started with fluorescence titrations of 25 µg/mL QDs with Cu(II) ions to select an optimal Cu(II) concentration for the competitive chemical tongue system. As shown in Fig. 1A, the fluorescence (FL) signal of QDs is gradually decreasing in the course of the experiment. Moreover, we noticed a redshift of QDs maximum emission (from 510 to approx. 520 nm) and an additional emission band in the range of 560–700 nm (Fig. 1A). These observations are in line with previous studies on quantum dots with the CdTe core [27–29] and are likely associated with the interaction of Cu(II) ions with the QDs surface, leading to changes in the charge transfer mechanism. As described in the literature, the application of Cu(II) ions could also be beneficial due to its susceptibility to reduction and distinct effects of Cu(II) and Cu(I) ions on the FL signal of QDs [27, 30, 31]. Such multifaceted changes in the FL spectrum induced by the Cu(II) addition could enrich the analytical response of QDs-based chemical tongue.

The abovementioned fluoresce quenching continued up to 5 µM Cu(II). However, the complete signal loss is not beneficial for the fabricated chemical tongue. First, if analytes bind Cu(II) ions, their removal from the surface of the nanocrystal might not guarantee the expected fluorescence recovery effect. Second, if analytes cause further fluorescence quenching of QDs, tracking these changes may not be possible. Therefore, the 4 µM Cu(II) concentration was selected for further analysis.

In the next step, we performed kinetic measurements to determine the time necessary to achieve a stable signal (Fig. 1B). After 30 min, a slight decrease in the fluorescence of the QDs/Cu(II) mixture can be observed. After 60 min, the FL signal remains at the same level; therefore, a 1-h incubation of Cu(II) ions with QDs was introduced to the protocol as a step proceeding with the addition of analytes.

### Multispectral fluorescence results

To evaluate the short-length peptide sensing capabilities of a chemical tongue competitive assay, seven of the most commonly studied N-terminal sequences of Aβ peptides were selected. Abbreviations used in this work, sequences, peptide charges without Cu(II), and charges of the Cu(II)/Aβ complexes for the 1:1 molar ratio, as well as the conditional binding constants of Cu(II)/Aβ at pH 7.4, are presented in Table 1.

**Table 1:**
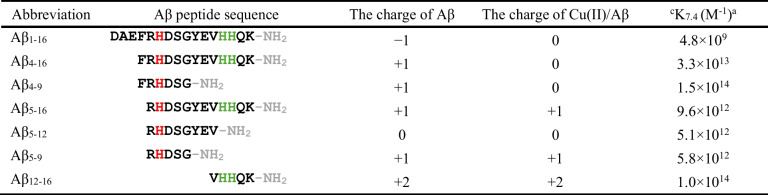
Amino acid sequences of the studied Aβ peptides, the charge of the peptide chain without Cu(II), the charge of Cu(II)/Aβ complexes for the 1:1 molar ratio, and the literature values of conditional Cu(II) binding constants at pH 7.4. The histidine residues in the sequences of Aβ peptides are marked in red, while the *bis*-His motif is marked as green

^a^The conditional binding constant of Cu(II) to Aβ peptides estimated for 1 mM concentration of reagents at pH 7.4 based on the literature data [24–26, 32]

**Fig. 2:**
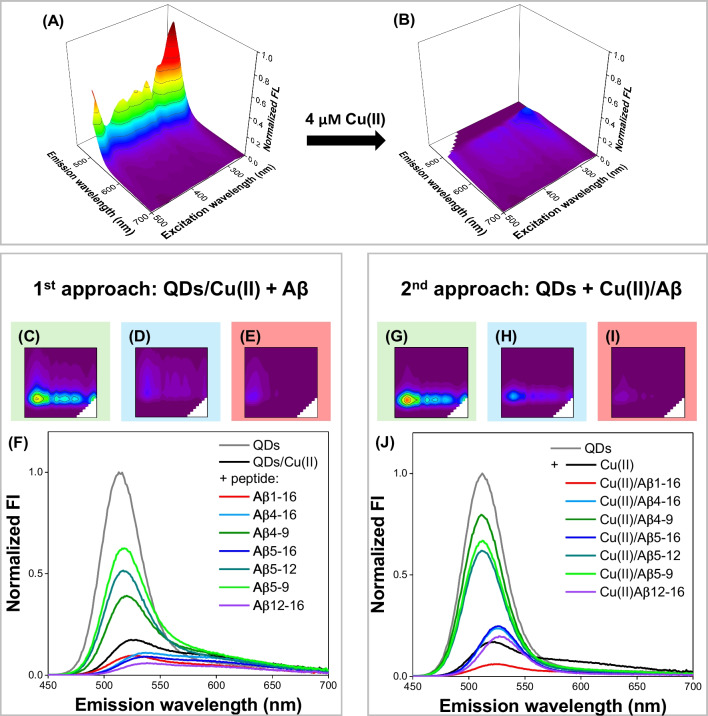
Comparison of the EEM fluorescence response obtained using the 1st and 2nd approach. EEM spectra of **A** QDs and **B** QDs/Cu(II). EEM spectra of QDs/Cu(II)/Aβ mixtures obtained using **C**–**E** the 1st and **F**–**H** the 2nd approach. Fluorescence emission spectra acquired under optimal excitation conditions (*λ*_ex_ : 290 nm) in the **F** 1st and **J** the 2nd approach. The spectra shown in Fig. 2F, J were prepared based on EEM data

We applied two protocols in the recognition of Aβ peptides. In the first one, Aβ peptides were introduced into the previously prepared QDs/Cu(II) mixture (the 1st approach: QDs/Cu(II) + Aβ). In the second one, previously prepared Cu(II)/Aβ mixtures were introduced to QDs solution (the 2nd approach: QDs + Cu(II)/Aβ). The comparison of multispectral fluorescence measurement results obtained using these approaches is shown in Fig. 2. Excitation-emission matrices acquired for all analytes are available in Electronic Supplementary Information (ESI), Fig. S1 and Fig. S2.

### 1^st^ approach: QDs/Cu(II) + Aβ

When Aβ peptides were introduced into the previously prepared QDs/Cu(II) mixture, three types of changes in their EEM fluorescence response were observed: (i) the signal recovery with a blueshift of emission maximum for Aβ_4-9_, Aβ_5-9_, and Aβ_5-12_; (ii) the further quenching of the fluorescence with a redshift of the signal maximum for Aβ_4-16_, Aβ_5-16_, and Aβ_12-16_; and (iii) the further quenching of the signal without any shift for Aβ_1-16_ (see Fig. 2C–E for the exemplary EEMs and Fig. 2F for the emission spectra acquired at optimal excitation conditions).

The peptides from the first group contain the His residue at the second (Aβ_5-9_ and Aβ_5-12_) or the third position (Aβ_4-9_) in the peptide sequence (see Table 1), representing high-affinity Cu(II) binding sites known as the His-2 and His-3 motifs, respectively. Thus, the effect of the enhancement and a blueshift of the QDs’ fluorescence signal (from approx. 520 to 515 nm) is likely due to the removal of Cu(II) ions from the nanocrystal surface (see Fig. 2C, Fig. S1 in the ESI). In contrast, the addition of the peptides from the second group (Aβ_4-16_, Aβ_5-16_, and Aβ_12-16_) to QDs/Cu(II) caused not only the further quenching of the fluorescence but also a redshift of the signal maximum (from approx. 520 to 530 nm; see Fig. 2D, Fig. S1). Interestingly, besides the His-2 (Aβ_5-16_, Aβ_12-16_) or His-3 (Aβ_4-16_, Aβ_12-16_) motifs, these peptides also contain two adjacent histidine residues known as *bis*-His motif (see Table 1). In our previous work on Aβ peptide discrimination using anionic CdTe quantum dots, we showed that the positively charged Aβ peptides possessing the *bis*-His motif cause the quenching of QDs’ fluorescence with a redshift of the maximum emission. We suspected that this signal change resulted from aggregation induced by electrostatic forces and the affinity of Aβ peptides with *bis*-His motif to the cadmium-rich surface of the nanocrystal [10]. The effect of analogous QDs-Aβ interactions most likely also prevails for Aβ_4-16_, Aβ_5-16_, and Aβ_12-16_ upon their addition to QDs/Cu(II), even despite the presence of high-affinity Cu(II) binding motifs (His-2 and His-3), which guaranteed the removal of Cu(II) ions from the QDs surface evidenced by FL signal recovery in case of Aβ_4-9_, Aβ_5-9_, and Aβ_5-12_ (Fig. S3). This could also be explained by the 25-fold excess of Aβ over Cu(II), which coincides with a greater impact of QDs-Aβ than Cu(II)-Aβ interactions on the observed FL signal changes. The quenching of QDs/Cu(II) fluorescence, but without the redshift of the emission maximum, was observed upon the addition of Aβ_1-16_. Interestingly, another effect was noticed instead, i.e., almost complete loss of the FL signal in the excitation range of 340–500 nm (Fig. 2E). The lack of the emission band shift aligns with our previous results [10], where the electrostatic repulsion between the negatively charged Aβ_1-16_ and anionic surface of thiomalic acid–capped QDs weakened the QDs-Aβ interaction. The Cu(II) binding constant for Aβ_1-16_ is also a few orders of magnitude lower than for other studied Aβ peptides (see Table 1), with the primary Cu(II) binding site also embracing the *bis*-His motif [33]. Consequently, the Cu(II) removal from the QDs surface by Aβ_1-16_ is less probable than for other studied Aβ fragments. It is worth noting that EEM spectra of all samples obtained using 1st approach also differed in fluorescence intensities, in order QDs > Aβ_5-9_ > Aβ_5-12_ > Aβ_4-9_ > Cu(II)/QDs > Aβ_4-16_ > Aβ_1-16_ > Aβ_5-16_ > Aβ_12-16_ (from the sample with the highest fluorescence intensity to the lowest; see Fig. 2F).

### 2^nd^ approach: QDs + Cu(II)/Aβ

Once again, three effects in EEM spectra were distinguished when previously prepared Cu(II)/Aβ mixtures were introduced to QDs solution (2nd approach): (i) the fluorescence quenching without any significant spectral shift for the Cu(II)/Aβ_4-9_, Cu(II)/Aβ_5-9_, and Cu(II)/Aβ_5-12_; (ii) the fluorescence quenching with a redshift for Cu(II)/Aβ_4-16_, Cu(II)/Aβ_5-16_, and Cu(II)/Aβ_12-16_; and (iii) the most extensive fluorescence quenching with a redshift for Cu(II)/Aβ_1-16_ (Fig. 2G–J, Fig. S2 in the ESI). The lack of the spectral shift for samples containing Cu(II)/Aβ_4-9_, Cu(II)/Aβ_5-9_, and Cu(II)/Aβ_5-12_ (Fig. 2G–J) advocates for the weakest interactions, the nature of which may differ for individual species among this group. The almost identical emission spectra obtained for QDs after the addition of the Cu(II)/Aβ_4-9_ mixture or Aβ_4-9_ alone (see the comparison of different approaches in Fig. S3) suggest that the observed effect for this peptide is mostly due to their electrostatic interactions with anionic QDs (Table 1). For Cu(II)/Aβ_5-9_ and Cu(II)/Aβ_5-12_ mixtures, the intensity of the QDs’ fluorescence signal is lower than for the respective Aβ peptides alone (Fig. S3), indicating greater impact of Cu(II) ions presence on the acquired EEM fluorescence response. This could be associated with the more facilitated interaction of Cu(II) complexes of His-2 peptides (Aβ_5-9_, Aβ_5-12_) with the external molecules, as well as the faster Cu(II) exchange [25], compared to the His-3 peptide complex represented by Cu(II)/Aβ_4-9_ [26].

The fluorescence quenching with a redshift noted in the presence of Cu(II)/Aβ_4-16_, Cu(II)/Aβ_5-16_, and Cu(II)/Aβ_12-16_ indicates their stronger interactions with QDs (Fig. 2H). All peptides from this group comprise the *bis*-His motif. Therefore, the interaction of their positively charged C-terminus embracing the *bis*-His motif with anionic QDs may be a leading force in the QD’s fluorescence signal changes. This assumption is supported by an almost identical response upon the addition of the Cu(II)/Aβ_12-16_ mixture and the Aβ_12-16_ alone to QDs (see Fig. S3), where the high-affinity Cu(II) binding site of the His-3 motif is within the *bis*-His motif (Table 1). By analogy to another His-3 complex, Cu(II)/Aβ_4-9_, the fully occupied Cu(II) binding sites in the equatorial plane of the Cu(II)/Aβ_12-16_ complex hinder the interactions of Cu(II) ions with other molecules, and the excess of the Aβ_12-16_ peptide mostly interacts with QDs. On the other hand, the primary role of the QDs-Aβ interactions also aligns with the same fluorescence signal intensity for Cu(II)/Aβ_4-16_ and Cu(II)/Aβ_5-16_, where the *bis*-His motif and high-affinity Cu(II) binding His-2 and His-3 motifs are separated (see fluorescence spectra at *λ*_ex_ of 290 nm in Fig. 2J). For these complexes, the *bis*-His motif is not occupied by Cu(II) ions. Thus, the Cu(II) complex could interact with QDs through the positively charged peptide C-terminus with the *bis*-His motif, whereas Cu(II) ions at the N-terminus may additionally affect the QDs’ fluorescence signal, leading to further fluorescence quenching, as shown in Fig. S3.

The sample containing the Cu(II)/Aβ_1-16_ mixture was characterized by the lowest fluorescence intensity in the group studied in the second approach (Fig. 2I). The decrease of the QD’s fluorescence signal upon the addition of the Cu(II)/Aβ_1-16_ mixture was much more pronounced than for the peptide alone and even more noticeable than upon the addition of Aβ_1-16_ to the preincubated QDs/Cu(II) in the first approach (Fig. S3). It could be connected with the weakest Cu(II) affinity to Aβ_1-16_ among other studied Aβ peptides, which could favor the FL signal quenching by Cu(II) ions. The similar redshift of the emission band for the Cu(II)/Aβ_1-16_ mixture and Cu(II) ions, but not for the Aβ_1-16_ alone, supports this hypothesis (see Fig. S3). The sequence of fluorescence intensity of samples measured using the 2nd approach is as follows: QDs > Aβ_4-9_ > Aβ_5-9_ > Aβ_5-12_ > Aβ_4-16_ ≈ Aβ_5-16_ > Aβ_12-16_ > Cu(II)/QDs > Aβ_1-16_ (Fig. 2J).

### Identification of Aβ peptides

**Fig. 3:**
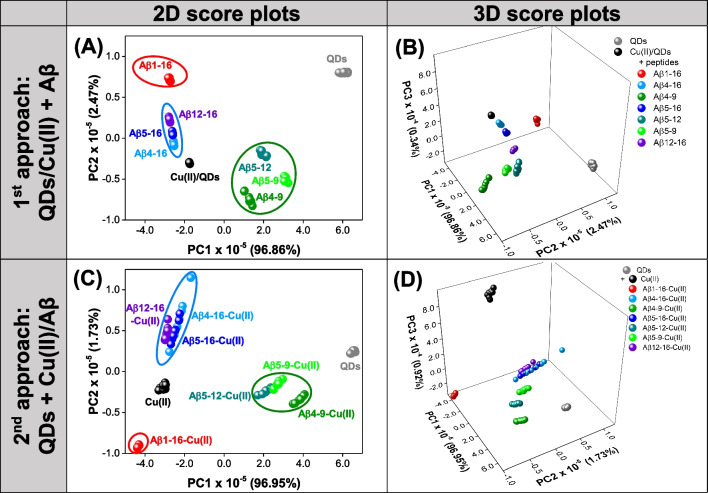
The score plots of PCA models developed using EEM fluorescent data obtained with **A**, **B** the 1st approach: QDs/Cu(II) + Aβ and **C**, **D** the 2nd approach: QDs + Cu(II)/Aβ. **A**, **C** Two-dimensional score plots. **B**, **D** Three-dimensional score plots

To explore the potential of two studied approaches (1st approach: QDs/Cu(II) + Aβ, 2nd approach: QDs + Cu(II)/Aβ) to perform chemical tongue competitive assay for Aβ peptide discrimination, we first processed obtained EEM spectra by unfolded principal component analysis (PCA). PCA is one of the most important and powerful chemometric methods used in analytical chemistry, which aims to determine a set of new directions in multidimensional space, i.e., principal components (PCs), describing the maximum variance of the original data set. The coordinates of newly created PCs can be graphically presented in score plots to assess the similarities and differences between investigated samples, which can be helpful in evaluating whether the original data contains information useful for their discrimination. In turn, the interpretation of the relationship between samples’ grouping in the PC space and the original data is possible due to a loadings plot investigation showing the contribution of the original variables to the following PCs [34]. PCA models with three PCs were developed based on unfolded EEM data acquired with the 1st and 2nd approach, and the resulting score plots are presented in Fig. 3. The loadings plots are presented in Fig. S4 available in Electronic Supplementary Information.

PCA model designated using EEM fluorescence data obtained with 1st approach (QDs/Cu(II) + Aβ) allows for differentiation of all measured samples in two-dimensional (Fig. 3A) and three-dimensional (Fig. 3B) principal component space, as the samples are grouped in well-separated clusters. In addition, the samples containing Aβ peptides inducing similar changes in the EEM spectrum of QDs/Cu(II) are located close to each other in PC1-PC2 space (Fig. 3A). The samples characterized by higher fluorescence intensity than QDs/Cu(II) control (QDs, Aβ_5-9_, Aβ_5-12_, Aβ_4-9_) exhibit positive scores on PC1. In turn, for QDs/Cu(II), Aβ_4-16_, Aβ_5-16_, Aβ_12-16_, and Aβ_1-16_, negative scores on PC1 are noted. The order of sample grouping in relation to PC1, as well as the loadings plot on PC1, indicates that it describes the effect of fluorescence quenching in the entire spectral range (see Fig. S4A in the ESI). The information included in PC2 allows to further differentiate QDs, Aβ_1-16_, Aβ_12-16_, with positive score values, Aβ_5-16_, Aβ_4-16_ with PC2 scores ≈ 0, and Aβ_5-12_, Cu(II)/QDs, Aβ_5-9_, Aβ_4-9_. The loadings on PC2 are negative in the excitation range of 270–500 nm and emission range of 560–650 nm, i.e., the spectral range in which the baseline changes related to QDs/Cu(II) interactions occur (see Fig. 2A, B). The positive loadings on PC2 are observed for *λ*_ex_ of 250–340 nm and *λ*_em_ of 490–540 nm, where the maximum signal of QDs in the EEM spectrum can be observed (Fig. S4B). The shape of PC2 loadings plot in this spectral range suggests that it may also be describing the effect of loss of fluorescence in the excitation range of 340–500 nm, which is also reflected by positive values on PC2 for Aβ_1-16_ exhibiting this effect (see Fig. 2E). Therefore, the grouping of samples along PC2 direction is the result of three effects: changes occurring within the baseline, changes in signal intensity in the range of the QDs emission maximum, and fluorescence decay at higher excitation wavelength (Fig. 3A). It must be noted that the clusters of Aβ_4-16_, Aβ_5-16_, and Aβ_12-16_ are very close to each other when only PC1-PC2 space is considered. Since the analysis of the loadings on PC3 showed that it contains complementary, valuable information for the differentiation of the samples (Fig. S4C), the clustering against PC3 was also investigated (Fig. 3B). As expected, better separation of Aβ_4-16_, Aβ_5-16_, and Aβ_12-16_ was achieved in three-dimensional PCA space. QDs/Cu(II), Aβ_4-16_, Aβ_5-9_, Aβ_1-16_, and Aβ_5-16_ exhibit positive scores on PC3, while for Aβ_5-12_, Aβ_12-16_, and Aβ_4-9_, PC3 scores are negative. The highest positive loadings on PC3 were observed for an excitation range of 250–340 nm and an emission range of 520–700 nm, which suggests that the shifts of the emission maximum have a large impact on this principal component. In addition, negative PC3 loadings can be distinguished for *λ*_ex_ of 400–500 nm and *λ*_em_ of 520–550 nm (Fig. S4C). Indeed, EEMs obtained with 1st approach (QDs/Cu(II) + Aβ) differ in the fluorescence signals in this spectral range, which, as can be seen in Fig. 2F, provides additional information differentiating the samples.

In the PCA model developed based on EEM fluorescence data acquired using the 2nd approach (QDs/Cu(II) + Aβ), similarly to the previous case, samples were clustering against PC1 according to their fluorescence intensity in the entire measured spectral range. The samples containing only QDs, as well as Aβ_4-9_, Aβ_5-9_, and Aβ_5-12_ quenching the FL signal of QDs to the least extent, are characterized by positive PC1 scores. As the fluorescence intensity of the sample decreases, scores on PC1 also decrease; thus, the samples with Aβ_4-16_, Aβ_5-16_, Cu(II), and Aβ_1-16_ exhibit negative PC1 values (Figs. 2J and 3C). Once again, the analysis of PC1 loadings confirms that it describes the change in fluorescence intensity of the measured samples over the entire EEM (Fig. S4D). It must be noted that due to the similarity of FL signal intensity in the case of samples containing Aβ_4-16_ and Aβ_5-16_, they are not distinguishable when only PC1 is considered. Moreover, the slight overlap of objects representing samples with Aβ_5-9_ and Aβ_5-12_ is also observed. PC2 contains the information related to shifts of the emission maximum as evidenced by the loadings plot in Fig. S4E in the ESI. The samples containing peptides inducing the redshift (Aβ_4-16_, Aβ_5-16_, Aβ_12-16_) are characterized by PC2 scores > 0, while for samples of Aβ_4-9_, Aβ_5-9_, and Aβ_5-12_, PC2 scores < 0 (Fig. 3C). Although PC3 also contains useful information, as it describes the changes in the baseline in the emission range of 560–650 nm (Fig. S4F), it mainly differentiates samples with Cu(II) ions from QDs control and the samples containing Aβ peptides (Fig. 3D). It is not surprising considering the method of sample preparation, in which QDs-Cu(II) interaction is highly hindered due to the excess of Aβ in relation to Cu(II) ions.

Unfolded partial least square–discriminant analysis (PLS-DA) was used in the next step to compare the efficiency of Aβ peptide discrimination using both investigated approaches. PLS-DA is a supervised chemometric method within which latent variables (LVs) are established, describing maximum covariance between original variables (e.g., spectral information describing tested samples) and the class membership of these samples [35]. Before PLS-DA model establishment, the data matrices previously applied to PCA modeling were randomly split into train set and test set, which were used for calibration and independent validation of the model, respectively. To compare the performance of the obtained models, four quality performance metrics were determined, i.e., accuracy, sensitivity, precision, and specificity (see Table S1 in the ESI). Confusion matrices obtained for the train set and test set providing numbers of true negatives (TN), false negatives (FN), false positives (FP), and true positives (TP) were used for the calculation of quality performance metrics. The “most probable” rule was applied to determine the class membership of the samples. Thus, the sample was assigned to the class for which the highest probability was achieved. The quality performance metrics of PLS-DA models designated with EEM data obtained using investigated sample preparation protocols (1st approach: QDs/Cu(II) + Aβ, 2nd approach: QDs + Cu(II)/Aβ) are shown in Table 2, while the confusion matrices are presented in Figs. S5 and S6 in the ESI.

**Table 2:** Quality performance metrics of PLS-DA models developed with EEM fluorescent data acquired with the 1st (QDs/Cu(II) + Aβ) and the 2nd (QDs + Cu(II)/Aβ) approach

Approach	Stage	Performance metric	QDs	QDs/Cu(II)	Aβ_1-16_	Aβ_4-16_	Aβ_4-9_	Aβ_5-16_	Aβ_5-12_	Aβ_5-9_	Aβ_12-16_
**1st: QDs/Cu(II) + Aβ**	Calibration	Accuracy	100%								
		Sensitivity	1.00	1.00	1.00	1.00	1.00	1.00	1.00	1.00	1.00
		Precision	1.00	1.00	1.00	1.00	1.00	1.00	1.00	1.00	1.00
		Specificity	1.00	1.00	1.00	1.00	1.00	1.00	1.00	1.00	1.00
	Validation	Accuracy	100%								
		Sensitivity	1.00	1.00	1.00	1.00	1.00	1.00	1.00	1.00	1.00
		Precision	1.00	1.00	1.00	1.00	1.00	1.00	1.00	1.00	1.00
		Specificity	1.00	1.00	1.00	1.00	1.00	1.00	1.00	1.00	1.00
**2nd: QDs + Cu(II)/Aβ**	Calibration	Accuracy	89%								
		Sensitivity	1.00	1.00	1.00	0.60	1.00	0.40	1.00	1.00	1.00
		Precision	1.00	1.00	1.00	0.50	1.00	0.50	1.00	1.00	1.00
		Specificity	1.00	1.00	1.00	0.93	1.00	0.95	1.00	1.00	1.00
	Validation	Accuracy	96%								
		Sensitivity	1.00	1.00	1.00	0.67	1.00	1.00	1.00	1.00	1.00
		Precision	1.00	1.00	1.00	1.00	1.00	0.75	1.00	1.00	1.00
		Specificity	1.00	1.00	1.00	1.00	1.00	0.96	1.00	1.00	1.00

PLS-DA model developed based on EEM data obtained with 1st approach (QDs/Cu(II) + Aβ) allowed for perfect identification of all samples (Fig. S5 in the ESI), as evidenced by the accuracy of 100% for both train set and test set (model calibration and independent validation, respectively). Consequently, the sensitivity (the ability to correctly identify sample), precision (the ability to avoid wrong predictions in the class), and specificity (the ability to reject samples of other classes) for all samples were 1.00 (Table 2). Slightly worse results were obtained when the PLS-DA model was established based on EEM fluorescence data obtained with the 2nd approach (QDs + Cu(II)/Aβ). Nevertheless, a satisfactory accuracy of 89% was achieved during model calibration (train set), while 96% of samples of the test set were correctly classified (Table 2). The sensitivity, precision, and specificity of QDs, Cu(II)/QDs, Aβ_1-16_, Aβ_4-9_, Aβ_5-12_, Aβ_5-9_, and Aβ_12-16_ were 1.00, as all of the samples containing these peptides were correctly classified. The lower accuracy results from misclassification of samples containing Aβ_4-16_ and Aβ_5-16_ (Fig. S6 in the ESI), which could be expected based on PCA results (Fig. 3C, D). Therefore, a sensitivity of 0.60, precision of 0.50, and specificity of 0.93 was achieved for the Aβ_4-16_ classification for the train set, while for the test set, these parameters were 0.67, 1.00, and 1.00, respectively. In turn, for Aβ_5-16_, the sensitivity of 0.40 and 1.00 were obtained, and the precision was 0.50 and 0.75, while the specificity was 0.95 and 0.96 (for train and test set, respectively; Table 2).

In our previous work, we showed that it is possible to satisfactorily differentiate the investigated Aβ peptide fragments using a chemical tongue based only on thiomalic acid–capped CdTe quantum dots [10]. Therefore, we finally compared whether the introduction of Cu(II) ions into the sensing system improves the discriminatory abilities of Aβ peptides. For this purpose, we developed a PLS-DA model based on EEM data of previously measured samples containing only QDs with Aβ peptide and analyzed the resulting confusion matrices for the train set and test set (Fig. S7 in the ESI). Despite the correct assignment of all samples from the train set, three Aβ peptides were misclassified. Similar to the results obtained with the 2nd approach (QDs + Cu(II)/Aβ), samples containing Aβ_4-16_ and Aβ_5-16_ were misclassified among themselves. However, in addition, the Aβ_5-12_ peptide was wrongly recognized as Aβ_4-9_. This result confirms that even a slight modification of QDs-based chemical tongue by the addition of transition metal ions might be beneficial, especially for the discrimination of peptides exhibiting higher structural resemblance. However, it should be emphasized that the method of conducting the experiment might be crucial to obtain the maximum possible amount of discriminatory information, as evidenced by the superiority of results obtained with the 1st (Fig. S5) and the 2nd (Fig. S6) approach.

## Conclusions

A high structural similarity and small size of short-length Aβ forms significantly complicate the detection of these compounds using classical methods, such as immunoassays. We proposed an alternative method to recognize such (bio)analytes, where changes in the multispectral fluorescence response of QDs exposed to Aβ peptides are recorded, leading to unique chemical fingerprints. In this work, we showed that such differentiation of chemical fingerprints obtained for individual Aβ peptides could be significantly improved by introducing Cu(II) ions. This way, competitive interactions occur between QDs, Aβ peptide, and Cu(II) ions, influencing the nature of the obtained EEM spectra. However, the optimal discriminatory abilities of the sensing system require a careful evaluation of the Aβ sample preparation protocol. In the case of the recognition of short-length Aβ forms, the preincubation of QDs with Cu(II) ions was beneficial for the peptide discrimination in contrast to another approach tested, where Cu(II) ions were firstly added to Aβ peptides. This potentially versatile method could be further modified by employing additional metal ions or quantum dots to detect other physiologically important peptides and (bio)analytes. Given an excellent recognition of such similar short-length Aβ peptides using QDs preincubated with Cu(II) ions, multispectral fluorescence, and chemometric methods, we plan to optimize further this approach for the detection of Aβ peptides of various lengths and sequences as a promising tool for Alzheimer’s disease diagnosis.

## Supplementary Information

Below is the link to the electronic supplementary material.

Supplementary file1 (DOCX 4.36 MB)

## Data Availability

Data are available in Supplementary Information and upon request.
