# Supplementary material for: Tuning multispectral fluorescence quantum dot–based identification of short-length amyloid β peptides by applying Cu(II) ions
Source: Mikrochim Acta. 2024 Oct 26;191(11):700. doi: 10.1007/s00604-024-06764-9 (PMC11512857; doi:10.1007/s00604-024-06764-9)
Supplement: Supplementary file 1 — Supplementary file1 (DOCX 4.36 MB) [file 604_2024_6764_MOESM1_ESM.docx]

**Electronic Supplementary Information
for
Tuning multispectral fluorescence quantum dots-based identification of short-length Aβ peptides by applying Cu(II) ions**

Klaudia Głowacz*, Weronika Tokarska, Anita Olechowska, Nina E. Wezynfeld*, Patrycja Ciosek-Skibińska*

Chair of Medical Biotechnology, Faculty of Chemistry, Warsaw University of Technology, Noakowskiego 3, 00-664 Warsaw, Poland

*Correspondence: [klaudia.glowacz@pw.edu.pl](mailto:klaudia.glowacz@pw.edu.pl) (KG), [nina.wezynfeld@pw.edu.pl](mailto:nina.wezynfeld@pw.edu.pl) (NEW), [patrycja.ciosek@pw.edu.pl](mailto:patrycja.ciosek@pw.edu.pl) (PCS)

## Multispectral fluorescence measurements

|  |  |  |
| --- | --- | --- |
|  |  |  |
|  |  |  |

**Figure S1**. EEM fluorescence spectra obtained using 1^st^ approach: QDs/Cu(II) + Aβ. The concentration of QDs was 25 µg/mL, Cu(II) ions was 4 µM and Aβ peptide was 100 µM. The spectra were acquired within excitation range of 250-500 nm, and emission of 450-700 nm.

| **** | **** | **** |
| --- | --- | --- |
| **** | **** | **** |
| **** | **** | **** |

**Figure S2**. EEM fluorescence spectra obtained using 2^nd^ approach: QDs + Cu(II)/Aβ. The concentration of QDs was 25 µg/mL, Cu(II) ions was 4 µM and Aβ peptide was 100 µM. The spectra were acquired within excitation range of 250-500 nm, and emission of 450-700 nm.

**
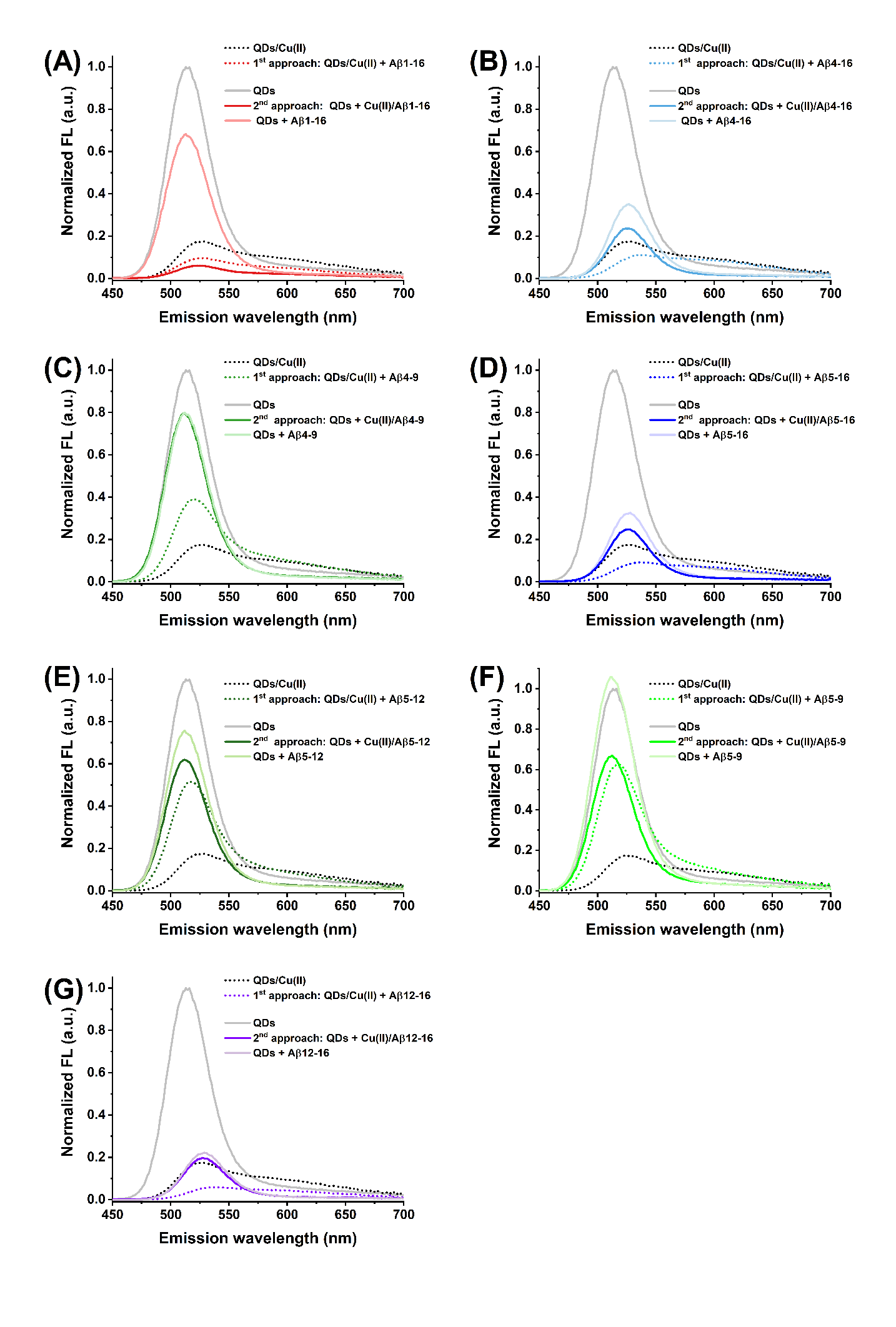
Figure S3.** The comparison of fluorescence emission spectra under optimal excitation conditions (λ_ex_ = 290 nm)
for a given Aβ peptide and different experimental approaches: the 1^st^ approach: QDs/Cu(II) + Aβ, where the Aβ peptide was added to the pre-incubated QDs/Cu(II) mixture, the 2^nd^ approach: QDs + Cu(II)/Aβ, where the previously prepared Cu(II)/Aβ mixture was added to QDs, and the previously studied approach (QDs + Aβ), where the Aβ peptide was added to QDs [1]. The concentration of reagents for all approaches were as follows: 25 µg/mL QDs, 4 µM Cu(NO_3_)_2_, 100 µM Aβ peptide in 50 mM HEPES pH 7.4.

## Chemometric analysis

| **(A)** | **PC1** | **PC2** | **PC3** |
| --- | --- | --- | --- |
| **1^st^ approach:**  **QDs/Cu(II) + Aβ** |  | **(C)**  **(B)** |  |
| **2^nd^ approach:**  **QDs + Cu(II)/Aβ** | **(D)** | **(F)**  **(E)** |  |

**Figure S4**. The loadings plots of PCA models developed using EEM fluorescent data obtained with (A-C) the 1^st^ approach: QDs/Cu(II) + Aβ and (D-F) the 2^nd^ approach: QDs + Cu(II)/Aβ. (A, D) PC1. (B, E) PC2. (C, F) PC3.

**Table S1.** Definitions of quality performance metrics used to assess the quality of PLS-DA models. (A) accuracy, (B) sensitivity, (C) precision, (D) specificity. TN – true negatives, FN – false negatives, FP – false positives, TP – true positives.

**(D)**

**(C)**

**(B)**

**(A)**

| $accuracy= \frac{(TP+TN)}{(TP+TN+FP+FN)}$ | $sensitivity= \frac{TP}{(TP+FN)}$ |
| --- | --- |
| $precision= \frac{TP}{(TP+FP)}$ | $specificity= \frac{TN}{(TN+FP)}$ |

**Figure S5**. Confusion matrixes of PLS-DA model developed using EEM fluorescent data obtained with the 1^st^ approach: QDs/Cu(II) + Aβ. (A) Calibration stage using train set. (B) Validation stage using independent test set.

**Figure S6**. Confusion matrices of PLS-DA model developed using EEM fluorescent data obtained with the 2^nd^ approach: QDs + Cu(II)/Aβ. (A) Calibration stage using train set. (B) Validation stage using independent test set.

**Figure S7**. Confusion matrices of PLS-DA model developed using EEM fluorescent data obtained previously and presented in [1], i.e., QDs + Aβ approach. (A) Calibration stage using train set. (B) Validation stage using independent test set.

[1] K. Głowacz, M. Drozd, W. Tokarska, N.E. Wezynfeld, P. Ciosek-Skibińska, Quantum dots-based “chemical tongue” for the discrimination of short-length Aβ peptides, Microchim. Acta. 191 (2024) 1–7. https://doi.org/10.1007/s00604-023-06115-0.
